# Changes in prescribed medicines in older patients with multimorbidity and polypharmacy in general practice

**DOI:** 10.1186/s12875-018-0825-3

**Published:** 2018-07-28

**Authors:** Fiona von Buedingen, Marc S. Hammer, Andreas D. Meid, Walter E. Müller, Ferdinand M. Gerlach, Christiane Muth

**Affiliations:** 1 Institute of General Practice, Johann Wolfgang Goethe University, Theodor-Stern-Kai 7, 60590 Frankfurt, Main Germany; 2 Department of Clinical Pharmacology and Pharmacoepidemiology, University of Heidelberg, Im Neuenheimer Feld 410, 69120 Heidelberg, Germany; 3 Pharmacological Institute for Natural Scientists, Johann Wolfgang Goethe University, Max-von-Laue-Str. 9, 60438 Frankfurt am Main, Germany

**Keywords:** Medication changes, Polypharmacy, Older adults, General practice, Multiple chronic conditions, Multimorbidity

## Abstract

**Background:**

Treatment complexity rises in line with the number of drugs, single doses, and administration methods, thereby threatening patient adherence. Patients with multimorbidity often need flexible, individualised treatment regimens, but alterations during the course of treatment may further increase complexity. The objective of our study was to explore medication changes in older patients with multimorbidity and polypharmacy in general practice.

**Methods:**

We retrospectively analysed data from the cluster-randomised PRIMUM trial (PRIoritisation of MUltimedication in Multimorbidity) conducted in 72 general practices. We developed an algorithm for active pharmaceutical ingredients (API), strength, dosage, and administration method to assess changes in physician-reported medication data during two intervals (baseline to six-months: ∆_1_; six- to nine-months: ∆_2_), analysed them descriptively at prescription and patient levels, and checked for intervention effects.

**Results:**

Of 502 patients (median age 72 years, 52% female), 464 completed the study. Changes occurred in 98.6% of patients (changes were 19% more likely in the intervention group): API changes during ∆_1_ and ∆_2_ occurred in 414 (82.5%) and 338 (67.3%) of patients, dosage alterations in 372 (74.1%) and 296 (59.2%), and changes in API strength in 158 (31.5%) and 138 (27.5%) respectively. Administration method changed in 79 (16%) of patients in both ∆_1_ and ∆_2_. Simvastatin, metformin and aspirin were most frequently subject to alterations.

**Conclusion:**

Medication regimens in older patients with multimorbidity and polypharmacy changed frequently. These are mostly due to discontinuations and dosage alterations, followed by additions and restarts. These findings cast doubt on the effectiveness of cross-sectional assessments of medication and support longitudinal assessments where possible.

**Trial registration.:**

1. Prospective registration: Trial registration number: NCT01171339; Name of registry: ClinicalTrials.gov; Date of registration: July 27, 2010; Date of enrolment of the first participant to the trial: August 12, 2010.

2. Peer reviewed trial registration: Trial registration number: ISRCTN99526053; Name of registry: Controlled Trials; Date of registration: August 31, 2010; Date of enrolment of the first participant to the trial: August 12, 2010.

**Electronic supplementary material:**

The online version of this article (10.1186/s12875-018-0825-3) contains supplementary material, which is available to authorized users.

## Background

General practitioners (GPs) are confronted with older patients with multiple chronic diseases in up to 80% of their consultations, i.e. patients with multimorbidity [1, 2]. Multimorbidity is strongly associated with polypharmacy, most commonly defined as the regular use of five or more drugs [3]. Polypharmacy increases the risk of inappropriate prescriptions from drug misuse (e.g., drug-drug interactions, drug-disease interactions, and inappropriate dosages) and drug selection (overuse and underuse), and may result in hospitalisations, falls and related injuries, decreased quality of life, cognitive and physical dysfunction, loss of autonomy, and increased mortality as well as preventable health care spending [4–12]. The indiscriminate use of disease-specific treatment guidelines for patients suffering from multimorbidity and uncoordinated treatment by multiple physicians are drivers of polypharmacy and lead to complex medication regimens involving multiple drugs [13–15]. However, medication adherence in patients is inversely correlated with the number of drugs and the complexity of the medication regimen [16]. The higher the number of drugs and doses per day, pills per dosage, different administration methods and specific recommendations related to drug use, such as dietary recommendations, the greater is treatment complexity and burden, and the patient’s inability to cope [14, 17, 18].

Medication changes over time may further increase treatment complexity because patients develop routines that enable them to manage their daily medication regimens, and adjusting them can be very troublesome for the chronically ill [19, 20]. On the other hand, medication changes often reflect patients’ preferences, particularly when conditions are symptomatic. The assessment of patient priorities and shared decision making when evaluating and changing medication in older patients with multimorbidity have been identified as important [21–23]. From the physician’s perspective, indications that an established drug regimen should be changed may be related to the development of new symptoms or worsening existing conditions, suspected medication-related adverse effects and because the treatment does not benefit the patient to the degree expected [24]. Particularly in patients with multimorbidity, evidence on effective treatment strategies is often lacking, and flexible and individualised pharmacotherapy is needed to meet their needs and preferences. As a result, GPs often rely on “hunches or best guesses” when deciding how to treat their patients [25]. Although, GPs frequently prefer to maintain the status quo once a patient appears to be stable [25], and are frequently reluctant to make changes to complex regimes, even if a potential problem has been identified [26, 27], their “hunches and best guesses” may lead to constant adaptation and frequent medication changes. Moreover, profound changes in a medication regimen are frequently made when patients are hospitalised and immediately thereafter. Newly introduced medication has not reached its steady state at discharge because inpatient care is frequently shorter than four to five half-lives of prescribed drugs, so that effectiveness and side-effects have not be properly assessed in hospital [28–30]. As variable discount contracts between pharmaceutical companies and statutory health insurers dictate the use of generic medicaments in Germany, changes may also be made in an attempt to reduce costs, rather than for medical reasons. Available consultation times apparently also influence prescribing behaviours. An average consultation time of less than seven minutes has been shown to be related to higher prescribing rates, whereas a consultation time of nine minutes or longer is associated with lower rates [31].

While all these determinants and consequences of prescribing behaviour and regimen complexity have been investigated in numerous cross-sectional studies, much less is known about how often medication regimens are changed over time, and what kind of changes older patients with multimorbidity and polypharmacy are faced with and have to manage. Improved insights into the number and nature of medication changes in this population may help strategies to be developed that avoid unnecessary medication changes. The goal of this study was therefore to explore changes in prescribed medication in older patients with multimorbidity and polypharmacy in primary care over a nine-month period.

## Methods

### Setting and population

We retrospectively assessed data from the pragmatic cluster-randomised controlled PRIMUM trial (PRIoritisation of MUltimedication in Multimorbidity) with the general practice as the unit of randomisation. The methods used in the PRIMUM trial are reported in detail elsewhere [32]. Briefly, the PRIMUM study evaluated the effectiveness of a complex intervention in improving medication appropriateness in comparison to usual care. The complex intervention consisted of four elements: the practice-based health care assistant (1) conducted a checklist-based interview with patients on medication-related problems and (2) reconciled their medications. (3) Assisted by a computerised decision-support system, the general practitioner optimised medication, and (4) discussed recommended changes with patients and adjusted medication regimens accordingly [32, 33]. Health care assistants in Germany receive less training than nurses, are comparable to certified medical assistants in the USA [34]. However, they have repeatedly and successfully carried out chronic care interventions that involve, for example, surveying patients by following protocols with fixed interview questions for conditions such as major depression, osteoarthritis and chronic heart failure, under the supervision of GPs [35–38].

Control practices continued with usual care. In usual care in Germany, patients regularly consult their GPs to discuss health problems, but, as GPs are not acting as ‘gate keepers’, may also contact ambulatory care specialists directly. Regular medication reviews were not conducted at the time of the study and health care for patients with chronic diseases has been characterized as fragmented and uncoordinated [39]. The presence of multiple conditions may have worsened the situation [40].

In total, 72 general practices located in the German state of Hesse participated in the PRIMUM trial. Of these practices, a random sample of 505 patients aged ≥60 years, with ≥3 chronic conditions for which they were treated with ≥5 drugs with systemic effects was included in the study. Patients were also required to be able to participate in telephone interviews, complete written questionnaires and provide written informed consent. Patients with cognitive impairment (Mini Mental State Exam < 26) and a life expectancy under 12 months were excluded. Patients’ degree of multimorbidity was assessed at baseline using the Cumulative Illness Rating Scale (CIRS) [41, 42].

### Measurement of medication changes

We assessed the number of changes to drugs, dosages and administration methods over a nine-month follow-up period. Data on prescribed medicines (active pharmaceutical ingredient (API) or trade name, dosage, and dosage form) were reported at baseline (T0), as well as six (T1), and nine (T2) months later. The first interval (∆_1_) was between baseline and the 6-month follow-up and the second interval (∆_2_) between the 6- and 9-month follow-ups.

At study visits, the attending health care assistant at the practice entered data on each participating patient into a paper-based case report form (CRF) and the GP checked the documentation before the CRF was sent to the study centre. At the study centre, a study assistant entered CRF data into an Access™ database where it was checked by another assistant. Based on an online database (“Gelbe Liste™”), an assistant coded the drugs according to API, strength, dosage form and a standardised package size of “N2” (generally containing the quantity of the drug required for 30 days of treatment) using the National Drug Code (PZN). PZN codes were converted into ATC codes, APIs, strengths, and dosage form automatically. Converted information was cross-checked against reported information for validation.

**Fig. 1:**
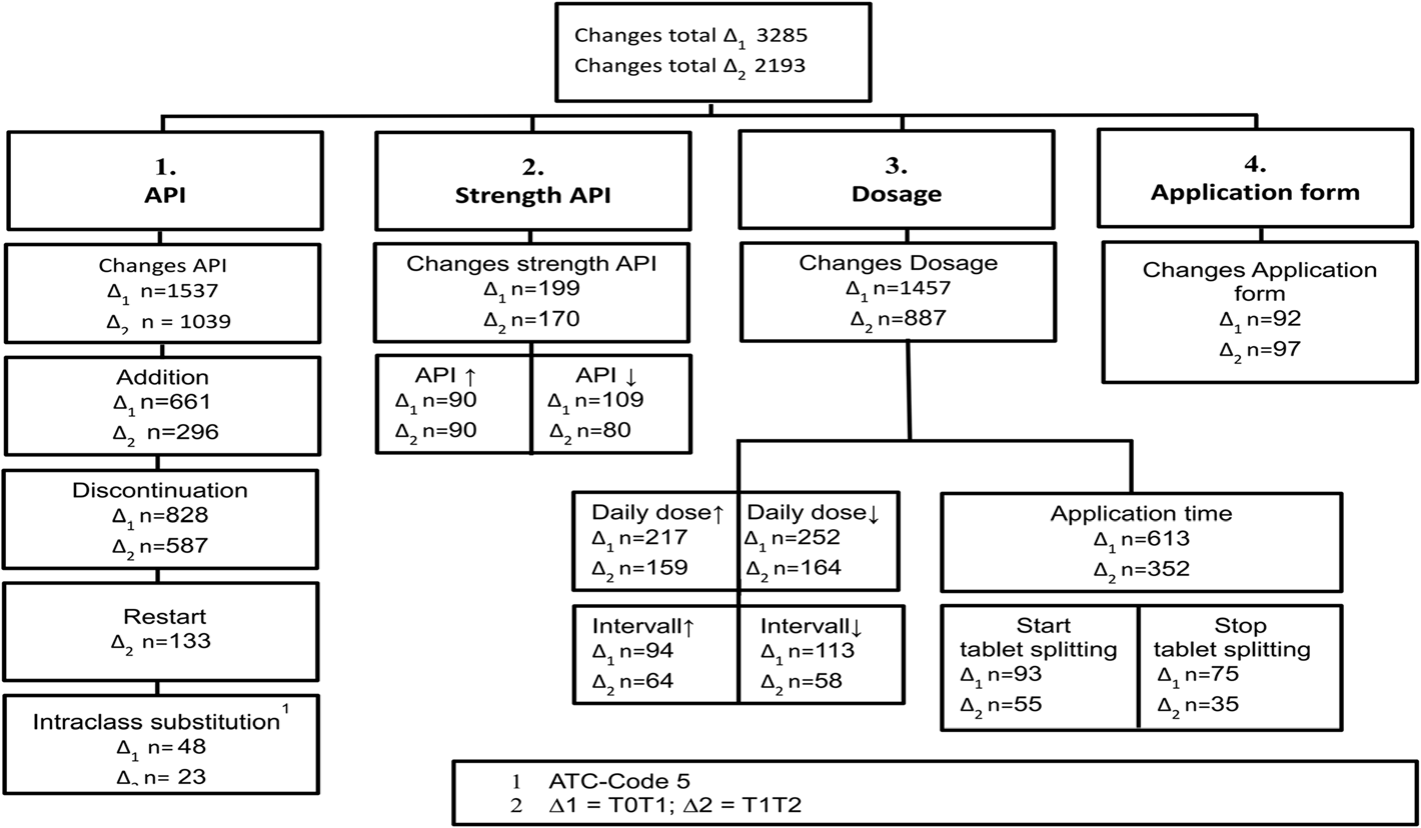
Medication Changes at Prescribing Level. API: active pharmaceutical ingredient. ^1^ATC-Code 5 digits. ATC: anatomical therapeutic chemical classification system. Interval ∆_1_: baseline to six-month follow-up; interval ∆_2_: six- to nine-month follow-ups

To explore medication changes in terms of medication usage, we adapted the algorithm suggested by Lam and co-workers [20]. We differentiated between changes (1) relating to API, (2) the strength of the API (amount of API per application unit e.g. tablet) (3) the dosage (the number of application units that make up the medication regimen) and (4) the administration method, reflecting the four main dimensions of a drug prescription (see Fig. 1).

API changes included the addition and discontinuation of drugs, restarts and intraclass-substitutions. Additions were medications that were present at one of the follow-up visits but were not reported before. Discontinuations referred to drugs that were mentioned previously but not reported at a later study visit. Restarts were drugs, which were discontinued between T0 and T1 but re-prescribed during the second interval (∆_2_). Intraclass substitutions were substitutions of a medicine by another drug of the same drug class, as reflected in the five-digit ATC-code. The strength of the API was changed if a higher or lower amount of the same API per unit of the medication was reported at one study visit compared to a previous one. The dosage was changed if the total daily dose (the amount of API per day) was increased or decreased, the application interval prolonged or shortened, and/or the application time altered between visits. The dosage was additionally modified when tablet splitting started or stopped. We measured changes to the administration method in accordance with the Medication Regimen Complexity Index (MRCI) [17]. CM assigned each of the 299 pharmaceutical dosage forms defined by the European Directorate for the Quality of Medicines & Health Care (EDQM) that were on the market at the time of the study to one of the 14 dosage forms used in the MRCI (immediate and extended release tablets, capsules, dragées, drops, liquids, effervescent tablets, sprays, ointments, plasters, metered-dose inhalers, syringes, suppositories, others). The classification was cross-checked by the clinical pharmacologist.

For medications with more than one active ingredient (combination drugs), we concentrated on changes in the active ingredient for the main indication of the drug. The main indication is represented by the three-digit ATC code describing the main therapeutic group for the particular medication. Changes in other active ingredients of combination drugs were not taken into consideration. FvB reviewed the medication changes of study participants. The algorithm was then applied electronically by MSH. Any disagreement was checked and corrected and the algorithm was refined where necessary.

### Statistical analysis

Data were analysed descriptively on a patient level and on the level of prescriptions using STATA version SE 13. For this purpose, we collected prescription data on each patient from the physicians at each one of the three study visits (T0, T1, T2). Changes from one study visit to the next were assessed for both intervals (∆_1_, and ∆_2_). A longitudinal prescription trajectory was developed for the prescriptions and study visits, with the patient’s first specific drug prescription as the reference (called *reference prescriptions* in the remainder of the article). The results are presented in absolute and relative numbers (percentages), means and standard deviations, or median and interquartile ranges (IQR).

The test for equal proportions was used to analyse changes and derive rate ratios between intervention and control groups. The Spearman correlation coefficient was used to analyse the relationship between the absolute numbers of medication changes in both time intervals.

The R software/environment version 3.3.2 (R Foundation for Statistical Computing, Vienna, Austria, 2016) was used for multivariate analyses and, in particular, to estimate and control for the intervention effect. Accounting for the clustering structure within GPs, a generalised linear mixed model was applied by the package *glmmADMB* [43]. Following an exploratory assessment of multi-collinearity and missing data patterns, the model was parameterised according to the independent variables *intervention status*, *age*, *sex* and respective changes in *number of previous hospital visits* at baseline, and the *Cumulative Illness Rating Scale (CIRS) summary score* between the follow-up visit and baseline. When modelling *total prescription* changes between study visits (T0, T1, T2), a negative binomial distribution provided the best fit to appropriately address overdispersion. Intra-cluster correlation (ICC) was obtained from the model in accordance with Aly and co-workers [44]. All tests were two-tailed. 95% confidence intervals (CI) were calculated, and *P* values < 0.05 were considered statistically significant.

## Results

Of the 72 participating GP’s, 41 worked in single-handed practices, and 31 in group practices. Their median age was 50 years (IQR 46.5–56), the majority was male (56.9%) and they had on average 23.5 years clinical experience (IQR 15.75–29).

We included 502 patients in our analyses (three had to be removed from the PRIMUM study population because they were younger than 60 at baseline), of whom 464 completed the study (see additional file 1). Their median age was 72 years (IQR 67–77), 52% of them were female (for patient’ characteristics in intervention and control group, see Table 1). At baseline and the two follow-up visits, the sum of all prescriptions was 11,719 and the total number of reference prescriptions was 4999. The most commonly prescribed therapeutic subgroups were agents acting on the renin-angiotensin system (ATC code C09: *n* = 514 prescriptions accounting for 10% of reference prescriptions), drugs used in diabetes (A10: *n* = 468, 9%), antithrombotic agents (B01: *n* = 409, 8%), drugs acting on the lipid metabolism (C10: *n* = 355, 7%) and beta blocking agents (C07: 352, 7%). See also Table 2.

**Table 1:** Baseline characteristics of patients

Characteristics (median, IQR)^a^	Intervention group (*n* = 252)	Control group (*n* = 250)	Total (*N* = 502)
Age	72 (69–77)	72 (66–77)	72 (67–77)
Female sex (n, %)	133 (53%)	129 (52%)	262 (52%)
CIRS sum score	7 (5–11)	7 (4–10)	7 (4–11)
Number of prescriptions	8 (6–10)	8 (6–9)	8 (6–9)
MRCI	24 (17–36)	24 (18–33)	24 (18–34)

*CIRS* cumulative illness rating scale, *MRCI* medication regimen complexity index

^a^if not stated otherwise

**Table 2:** Most commonly prescribed medicines

Chemical subgroup^b^ Most prevalent active pharmaceutical ingredient (API)^c^	Absolute and relative frequency of prescriptions^a^ (n, %)
Platelet aggregation inhibitors excl. Heparin (B01AC):	323 (6.5%)
Acetylsalicylic acid (B01AC06)	261 (5.2%)
Beta blocking agents, selective (C07AB):	297 (5.9%)
Bisoprolol (C07AB07)	135 (2.7%)
Metoprolol (C07AB02)	128 (2.6%)
HMG CoA reductase inhibitors (C10AA)	297 (5.9%)
Simvastatin (C01AA01)	256 (5.1%)
Proton pump inhibitors (A02BC)	231 (4.6%)
Omeprazole (A02BC01)	126 (2.5%)
Pantoprazole (A02BC02)	93 (1.9%)
ACE inhibitors, plain (C09AA)	218 (4.4%)
Ramipril (C09AA05)	139 (2.8%)
Dihydropyridine derivatives (C08CA)	208 (4.2%)
Amlodipine (C08CA01)	148 (3.0%)
Sulfonamides, plain (C03CA)	176 (3.5%)
Torasemide (C03CA04)	133 (2.7%)
Thyroid hormones (H03AA)	159 (3.2%)
Levothyroxine (H03AA01)	119 (2.4%)
Biguanides (A10BA)	131 (2.6%)
Metformin (A10BA02)	131 (2.6%)
Preparations inhibiting uric acid production (M04AA)	123 (2.5%)
Allopurinol (M04AA01)	120 (2.4%)
Angiotensin II antagonists, plain (C09CA)	117 (2.3%)
Candesartan (C09CA06)	40 (0.8%)
Low-Ceiling Diuretics, Thiazides, plain (C03AA)	97 (1.9%)
Hydrochlorothiazide (C03AA03)	97 (1.9%)
Selective beta-2-adrenoreceptor agonists (R03AC)	95 (1.9%)
Salbutamol (R03AC02)	46 (0.9%)

^a^Relative frequency: absolute numbers of prescriptions with regard to the total number of reference prescriptions (*N* = 4999)

^b^according to the 4th level, chemical subgroup (5-digit ATC code)

^c^according to the 5th level, chemical substance (7-digit ATC code)

### Medication changes at patient level

**Fig. 2:**
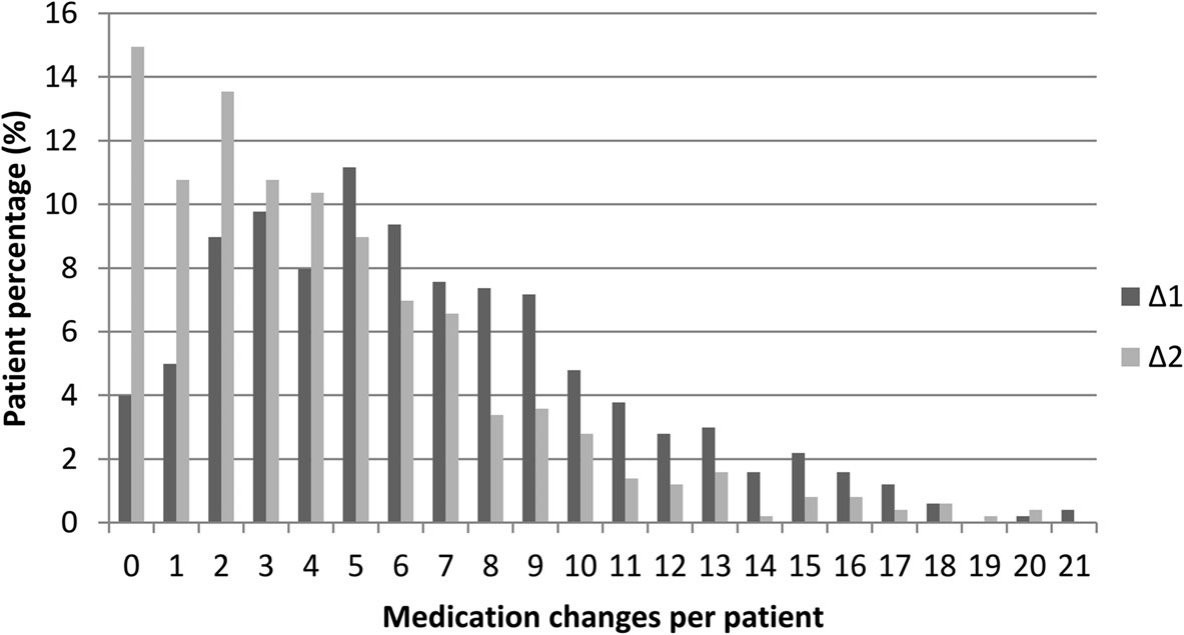
Medication Changes at Patient Level. Interval ∆1: baseline to six-month follow-up; interval ∆2: six- to nine-month follow-ups

Cumulatively, 99% of patients showed at least one medication change over the nine-month period with a median of 9.5 (IQR 6–15) changes per patient. The maximum of medication changes was 21 (in one patient) during the six months of Δ_1_ and 20 (in two patients) during the three months in Δ_2_. The mean number of changes per patient was 6.5 (STD 4.3) in ∆_1_ and 4.4 (STD 3.9) in ∆_2_. The total number of drugs per patient remained relatively constant (the median number of prescriptions was 8 at all study visits, while the IQR was 6 to 9 at T0 and T1 and 6 to 10 at T2). (See Fig. 2).

The most frequent changes occurred in the active ingredient, the subcategories of which were addition, discontinuation, intraclass substitution and restart (∆_2,_ only). The API was changed in 414 (83%) of the patients during ∆_1_ and 338 (67%) during ∆_2_ (in ∆_1_ and ∆_2,_ the mean number of API changes per patient were 3.1 and 2.1 respectively). We observed up to eight additions per patient in both periods. The mean number of added drugs per patient was 1.3 (STD 1.5) during ∆_1_ and 0.6 (STD 1.0) during ∆_2_. Patients had discontinued up to 15 drugs in ∆_1_ and 16 in ∆_2_, with a mean number of de-prescribed drugs per patient of 1.6 (STD 1.9) in ∆_1_ and 1.7 (STD 2.0) in ∆_2_.

Changes in the strength of the API occurred in 158 (32%) patients in ∆_1_ and 138 (28%) patients in ∆_2_ (in ∆_1_ and ∆_2,_ mean change per patient was 0.4 (STD 0.65) and 0.3 (STD 0.61) respectively). Dosages were altered in 372 (74%) patients in ∆_1_ and in 296 (59.2%) patients in ∆_2_. The mean number of dosage changes per patient was 2.9 (STD 2.87) and 1.8 (STD 2.21) respectively (Table 3).

**Table 3:** Medication changes at patient level

	Δ1 (baseline to 6-month follow-up)				Δ2 (6-month follow-up to 9-month follow-up)			
	Intervention group (IG) (n = 252)	Control group (CG) (n = 250)	Total (N = 502)	Proportion difference IG vs. CG (95% CI)	Intervention group (IG) (n = 252)	Control group (CG) (n = 250)	Total (N = 502)	Proportion difference IG vs. CG (95% CI)
API								
Addition	160 (63%)	153 (61%)	313 (62%)	0.02 (−0.07;0.11) *P* = 0.661	93 (37%)	88 (35%)	181 (36%)	0.02 (− 0.07;0.11) *P* = 0.761
Discontinuation	176 (70%)	161 (64%)	337 (67%)	0.05 (− 0.03;0.14) *P* = 0.229	119 (47%)	129 (52%)	248 (49%)	−0.04 (− 0.14;0.05) *P* = 0.373
Intraclass substitution	23 (9%)	18 (7%)	41 (8.2%)	0.02 (− 0.03;0.07) *P* = 0.532	14 (6%)	7 (3%)	21 (4%)	0.03 (− 0.01;0.07), *P* = 0.187
Restart of previously discontinued drugs	–	–	–	n.a.	51/176 (29%)	53/161 (30%)	104/337 (31%)	− 0.01 (− 0.08;0.07), *P* = 0.876
Strength of the API								
Increase	46 (18%)	36 (14%)	82 (16%)	0.04 (− 0.03;0.11) *P* = 0.295	45 (18%)	35 (14%)	80 (16%)	0.04 (−0.03;0.11), *P* = 0.29
Decrease	44 (17%)	49 (20%)	93 (19%)	−0.02 (− 0.09;0.05), *P* = 0.616	31 (12%)	37 (15%)	70 (14%)	− 0.02 (− 0.09;0.04), *P* = 0.492
Dosage								
Daily dosage increase	91 (36%)	80 (32%)	171 (34%)	0.04 (− 0.05;0.13), *P* = 0.38	67 (27%)	61 (24%)	128 (26%)	0.02 (−0.06;0.1), *P* = 0.646
Daily dosage decrease	106 (42%)	79 (32%)	185 (37%)	*0.1 (0.02;0.19)*, P = 0.019*	72 (29%)	58 (23%)	130 (26%)	0.05 (− 0.03;0.13), *P* = 0.203
Application interval shortened	55 (22%)	42 (17%)	97 (19%)	0.05 (− 0.02;0.12), *P* = 0.189	32 (13%)	20 (8%)	52 (10%)	0.05 (− 0.01;0.1), *P* = 0.114
Application interval prolonged	48 (19%)	35 (14%)	83 (17%)	0.05 (− 0.02;0.12), *P* = 0.161	28 (11%)	26 (10%)	54 (11%)	0.01 (−0.05;0.07), *P* = 0.91
Change in application time point	145 (58%)	155 (62%)	300 (60%)	− 0.04 (− 0.13;0.05), *P* = 0.353	98 (39%)	118 (47%)	216 (45%)	− 0.08 (− 0.17;0.01), *P* = 0.073
Pill splitting started	43 (17%)	35 (14%)	78 (16%)	0.03 (−0.04;0.1), *P* = 0.41	23 (9%)	19 (8%)	42 (8%)	0.02 (− 0.04;0.07), *P* = 0.648
Pill splitting stopped	30 (12%)	35 (14%)	65 (13%)	−0.02 (− 0.08;0.04), *P* = 0.571	13 (5%)	18 (7%)	31 (6%)	− 0.02 (− 0.07;0.03), *P* = 0.445
Administration method								
Change in administration method	47 (19%)	32 (13%)	79 (16%)	0.06 (− 0.01;0.13), *P* = 0.093	37 (15%)	42 (17%)	79 (16%)	−0.02 (− 0.09;0.05), *P* = 0.597

*API* active pharmaceutical ingredient

*Difference between intervention group and control group was statistically significant

There was no statistical correlation between medication changes in ∆_1_ and ∆_2_ with correlation coefficients indicating weak relationships, if any (Spearman correlation coefficient for overall medication changes between ∆_1_ and ∆_2_ was 0.321 and 0.005 between discontinuations in ∆_1_ and any changes in ∆_2_). The multivariate model showed that more medication changes occurred in the intervention group. We compared both groups and found 19% more medication changes in the intervention group (95% CI [0.3%; 41%], *P* = 0.0046), which was the only significant factor in the multiple regression model. It is noteworthy that an ICC of 0.146 was observed. Univariate analyses showed that the reduction in the dosage was slightly greater but significantly so in the intervention group compared to the control group but no further significant differences in the various types of medication changes were found between both groups (Table 3).

### Medication changes at prescribing level

The analysis of the medication included *N* = 4999 reference prescriptions. In total, we detected *N* = 5478 cumulative changes in these reference prescriptions during the nine-month follow-up. Figure 1 shows the distribution of changes in reference prescriptions, as determined using our algorithm. The mean number of changes per reference prescription was 1.09 (STD 1.2). The most frequently changed drugs in our older study population with multimorbidity were simvastatin (mostly, a change in the strength of API, an increase or decrease of dosage, a change in the application time and starting or stopping tablet splitting), low dose aspirin (addition, discontinuation and restart as well as changes in time of application), metformin (application interval and time, and decrease in dosage), omeprazole (intraclass substitution of pantoprazole or esomeprazole) and ramipril (strength of API and application interval; see Table 4). Most of the restarts (after previous discontinuation) were seen in low dose aspirin, metoprolol, HCT, ramipril and diclofenac.

**Table 4:** Most frequently changed drugs

Interval Δ_1_ (baseline to 6 month follow-up)				Interval Δ_2_ (6-month follow-up to 9-month follow-up)			
Intervention group		Control group		Intervention group		Control group	
Drug	Changes in Δ_1_ / total prescriptions at T0^a^ (n / N, %)	Drug	Changes in Δ_1_ / total prescriptions at T0^a^ (n / N, %)	Drug	Changes in Δ_2_ / total prescriptions at T1^a^ (n / N, %)	Drug	Changes in Δ_2_ / total prescriptions at T1^a^ (n / N, %)
Simvastatin	75 / 104 (72%)	Simvastatin	90 / 128 (70%)	Aspirin*	40 / 108 (37%)	Simvastatin	72 / 121 (60%)
Ramipril	70 / 67 (104%)	Aspirin*	58 / 126 (46%)	Omeprazole	39 / 48 (81%)	Aspirin*	50 / 122 (41%)
Metformin	63 / 61 (103%)	Metformin	49 / 57 (86%)	Simvastatin	38 / 107 (35%)	Metformin	39 / 50 (78%)
Amlodipine	63 / 76 (83%)	Allopurinol	43 / 47 (91%)	Ramipril	37 / 64 (56%)	Omeprazole	34 / 52 (65%)
Metoprolol*	59 / 69 (85%)	Amlodipine	40 / 57 (70%)	Metformin	34 / 60 (57%)	Torasemide	33 / 50 (66%)
Bisoprolol	58 / 62 (93%)	Pantoprazole	39 / 33 (118%)	Torasemide	34 / 57 (60%)	Metoprolol*	31 / 49 (63%)
Omeprazole	54 / 55 (98%)	Bisoprolol	39 / 65 (60%)	Pantoprazole	29 / 31 (93%)	Ramipril	29 / 58 (50%)
Aspirin*	53 / 105 (50%)	Metoprolol*	38 / 54 (70%)	Allopurinol	28 / 44 (64%)	Pantoprazole	28 / 29 (96%)
Torasemide	45 / 52 (87%)	Ramipril	38 / 54 (70%)	Metoprolol*	27 / 67 (40%)	Amlodipine	25 / 53 (47%)
Allopurinol	45 / 53 (85%)	Omeprazole	37 / 50 (74%)	Amlodipine	25 / 70 (36%)	Bisoprolol	24 / 62 (39%)
Levothyroxine	40 / 55 (73%)	Torasemide	37 / 55 (67%)	Bisoprolol	18 / 63 (29%)	Allopurinol	23 / 44 (52%)
Pantoprazole	37 / 30 (123%)	HCT	24 / 40 (60%)	Diclofenac	17 / 34 (50%)	Diclofenac	22 / 12 (183%)
Diclofenac	33 / 36 (92%)	Levothyroxine	23 / 55 (42%)	Ca + D3	16 / 14 (114%)	HCT	20 / 37 (54%)

^†^number of changed prescriptions during the interval per number of prescriptions at the begin of the interval, percentages > 100% indicate prescriptions with more than one change during the interval under investigation. API – active pharmaceutical ingredient, Aspirin* - low dose aspirin, Ca + D3 – combination of calcium and cholecalciferol (vitamin D3), HCT – hydrochlorothiazide, Metoprolol* - metoprolol-succinate

## Discussion

### Key findings

In our study population of older patients with multimorbidity and polypharmacy, more than three quarters of prescriptions was modified within six months (∆_1_) and another half in the three months thereafter (∆_2_). According to our algorithm, changes occurred in all categories, with changes in API (83%) the most common, and changes in administration method (16%) the least. The API was most often changed through discontinuation (in more than two thirds of the patients at least one drug was stopped during ∆_1_ and in almost half the patients during ∆_2_). However, our data also showed that at least one third of previously discontinued drugs were restarted (we could only measure restarts in the second period, ∆_2_). Furthermore, the number of dosage alterations was high and observed in 82% of patients. Despite such high levels of prescribing and de-prescribing, the total number of medications per patient remained remarkably constant. This finding supports the general view that drug numbers barely reflect prescription appropriateness [45, 46]. In order to optimise a patient’s medication, it can be warranted both to stop (i.e., de-prescribe) unnecessary drugs and to start taking indicated drugs [47].

On a prescription level, our research revealed that the most frequent modifications occurred in simvastatin and low-dose aspirin, which are both drugs that should be prescribed in relatively fixed doses in the treatment (or prevention) of chronic conditions. Changes in dosage were the most frequently observed modifications in both drugs. In the case of simvastatin, presumed or actual common side effects (myalgia) [48] may have encouraged the treating physician to replace the statin with an intermittent or pulsed dosing regimen [49], or to slowly increase doses in highly intolerant patients [50]. Restarts of metoprolol, ramipril, diuretics or diclofenac may have been decided when underlying health problems exacerbated and precluded a discontinuation or withdrawal symptoms occurred [51]. The intraclass substitution of pantoprazole for omeprazole may reflect the reduced likelihood of drug-drug interactions in pantoprazole [52]. Other frequent modifications, such as discontinuation or dosage alterations in diuretics or anti-diabetics are easier to interpret, but the reasons behind changes were not documented in our study.

### Comparison with literature

The basic pattern of medication changes in our study was consistent with a previous observation study on older patients with multimorbidity and polypharmacy which also identified frequent starting, stopping and dosage changes [20]. However, the proportion of patients whose medication regimen was modified was higher in our study than in the previous one, in which additions were observed in 61%, discontinuations in 58%, dose changes in 51% and restarts in 21% of patients [20]. The differences may reflect different study designs and data collection methods. While we collected primary data from GPs in a polypharmacy study on medication optimisation, Lam et al. analysed claims data [20]. In our analysis, the intervention slightly increased the rate at which changes were made in the intervention group. In de-prescribing trials in community-dwelling elderly patients, 47% [53] and 48% [54] of patients reported at least one discontinuation and the average was 4.2 per patient [53]. In studies conducted in nursing homes, drugs were withdrawn in 59% [55] and 63% [56] of patients (on average 4.4 and 2.8 per patient respectively). However, no other medication changes were reported in these studies.

### Strengths & Limitations

The robust design of the pragmatic cluster-randomised controlled PRIMUM trial was a major strength. This is particularly reflected in the random sample of patients per practice, the collection of primary data, the quality assurance and validation of medication data, the stepwise development and forward-backward validation of the algorithm to explore medication changes and its application by two independent researchers. This allowed a rich exploratory approach to the number and pattern of medication changes in older patients with multimorbidity and polypharmacy and the extension of the algorithm provided by Lam et al. to include the patient’s perspective [20]. However, the study design was also a limitation because the intention of the intervention was to identify and change inappropriate medication regimens. The vast majority of patients included in the PRIMUM study received appropriate prescriptions according to the Medication Appropriateness Index (MAI) [57] and reported good quality of life and functional status. The intervention had no effect on primary and secondary outcomes [32], but medication changes did slightly increase (according to the multivariate model, 19% more frequently in the intervention group, with slightly more dosage reductions). The MAI does not reliably detect underuse [58] and our ratings may have missed also inappropriateness. Furthermore, the PRIMUM study included three measuring points over a period of nine months, which resulted in a series of cross-sectional measurements rather than continuous documentation, so we may have missed a number of medication changes between study visits and our results may therefore be rather conservative.

### Implication

Our results illustrate that medication regimens in older patients with multimorbidity and polypharmacy are relatively unstable, and this has major implications. Firstly, future analyses should seek to discriminate appropriate from inappropriate, planned vs. ad hoc changes and examine changes in high-risk drugs, as these are more likely to be clinically significant for patients [59]. For clinicians and patients, it is of interest whether frequent medication changes increase longitudinal complexity and whether this affects patient adherence and health care costs in developing strategies to avoid unnecessary alterations in medication regimens in elderly, chronically ill patients with multimorbidity. Secondly, our findings pose fundamental methodological questions with respect to polypharmacy studies (no matter whether observational or interventional): how reliable are cross-sectional measurements of medication in this population, and what are suitable measurement intervals? Future studies should assess longitudinal changes so that researchers in the field can select appropriate study intervals.

## Conclusion

Medication regimens in older patients with multimorbidity and polypharmacy change frequently. Most of the changes are discontinuations and dosage alterations followed by additional prescriptions and restarts. Modifications also include drugs, such as statins and low-dose aspirins, that are intended for prescription in relatively fixed dosages in the treatment of chronic conditions. These findings cast doubt on the effectiveness of cross-sectional assessments of medication (appropriateness) and support longitudinal assessments where possible. Dedicated future studies should longitudinally assess changes in medication regimens and seek to explain them. They should also study their effect on patient adherence.

## Additional File

Additional File 1: Flowchart patient allocation PRIMUM Study. (PDF 340 kb)

## Abbreviations

API: Active pharmaceutical ingredient
ATC: Anatomical therapeutic chemical classification system
CIRS: Cumulative illness rating scale
CRF: Case report form
EDQM: European Directorate for the Quality of Medicines & Health Care
GP: General practitioner
IQR: Interquartile range
MRCI: Medication regimen complexity index
PRIMUM: PRIoritisation of MUltimedication in Multimorbidity
PZN: National Drug Code
STD: Standard deviation

## References

[1] Salisbury C, Johnson L, Purdy S, Valderas JM, Montgomery AA (2011). Epidemiology and impact of multimorbidity in primary care: a retrospective cohort study. Br J Gen Pract.

[2] van den Akker M, Buntinx F, Knottnerus J (1996). Comorbidity or multimorbidity: what's in a name. A review of literature. Eur J Gen Pract.

[3] Masnoon N, Shakib S, Kalisch-Ellett L, Caughey GE (2017). What is polypharmacy? A systematic review of definitions. BMC Geriatr.

[4] Campbell SE, Seymour DG, Primrose WR, Lynch JE, Dunstan E, Espallargues M, Lamura G, Lawson P, Philp I, Mestheneos E (2005). A multi-Centre European study of factors affecting the discharge destination of older people admitted to hospital: analysis of in-hospital data from the ACMEplus project. Age Ageing.

[5] Davies EC, Green CF, Taylor S, Williamson PR, Mottram DR, Pirmohamed M (2009). Adverse drug reactions in hospital in-patients: a prospective analysis of 3695 patient-episodes. PLoS One.

[6] Deandrea S, Lucenteforte E, Bravi F, Foschi R, La VC, Negri E (2010). Risk factors for falls in community-dwelling older people: a systematic review and meta-analysis. Epidemiology.

[7] Grimmsmann T, Himmel W (2009). Polypharmacy in primary care practices: an analysis using a large health insurance database. Pharmacoepidemiol Drug Saf.

[8] Hakkarainen KM, Gyllensten H, Jonsson AK, Andersson SK, Petzold M, Hagg S (2014). Prevalence, nature and potential preventability of adverse drug events - a population-based medical record study of 4970 adults. Br J Clin Pharmacol.

[9] Meid AD, Quinzler R, Groll A, Wild B, Saum KU, Schottker B, Heider D, Konig HH, Brenner H, Haefeli WE (2016). Longitudinal evaluation of medication underuse in older outpatients and its association with quality of life. Eur J Clin Pharmacol.

[10] Pirmohamed M, James S, Meakin S, Green C, Scott AK, Walley TJ, Farrar K, Park BK, Breckenridge AM (2004). Adverse drug reactions as cause of admission to hospital: prospective analysis of 18 820 patients. BMJ.

[11] Thomson LA, Winterstein AG, Sondergaard B, Haugbolle LS, Melander A (2007). Systematic review of the incidence and characteristics of preventable adverse drug events in ambulatory care. Ann Pharmacother.

[12] Viktil KK, Blix HS, Moger TA, Reikvam A (2007). Polypharmacy as commonly defined is an indicator of limited value in the assessment of drug-related problems. Br J Clin Pharmacol.

[13] Boyd CM, Darer J, Boult C, Fried LP, Boult L, Wu AW (2005). Clinical practice guidelines and quality of care for older patients with multiple comorbid diseases: implications for pay for performance. JAMA.

[14] Muir AJ, Sanders LL, Wilkinson WE, Schmader K (2001). Reducing medication regimen complexity: a controlled trial. J Gen Intern Med.

[15] Rambhade S, Chakarborty A, Shrivastava A, Patil UK, Rambhade A (2012). A survey on polypharmacy and use of inappropriate medications. Toxicol Int.

[16] Horne R, Weinman J, Barber N, Elliott R, Morgan M, Cribb A, I K: Concordance, adherence and compliance in medicine taking. *National Coordinating Centre for the Service Delivery and Organisation (NCCSDO)* 2005. URL: http://www.netscc.ac.uk/hsdr/files/project/SDO_FR_08-1412-076_V01.pdf; Accessed: 08/07/2018.

[17] George J, Phun YT, Bailey MJ, Kong DC, Stewart K (2004). Development and validation of the medication regimen complexity index. Ann Pharmacother.

[18] Stone VE, Hogan JW, Schuman P, Rompalo AM, Howard AA, Korkontzelou C, Smith DK (2001). Antiretroviral regimen complexity, self-reported adherence, and HIV patients' understanding of their regimens: survey of women in the her study. J Acquir Immune Defic Syndr.

[19] Haslbeck JW, Schaeffer D (2009). Routines in medication management: the perspective of people with chronic conditions. Chronic Illn.

[20] Lam KD, Miao Y, Steinman MA (2013). Cumulative changes in the use of long-term medications: a measure of prescribing complexity. JAMA Intern Med.

[21] Muth C, van den Akker M, Blom JW, Mallen CD, Rochon J, Schellevis FG, Becker A, Beyer M, Gensichen J, Kirchner H (2014). The Ariadne principles: how to handle multimorbidity in primary care consultations. BMC Med.

[22] NICE: Multimorbidity: clinical assessment and management. Multimorbidity: assessment, prioritisation and management of care for people with commonly occurring multimorbidity. NICE guideline NG56. National Institute for Health and Care Excellence. 2016. URL: https://www.nice.org.uk/guidance/ng56/evidence; Accessed: 08/07/2018. 27683922

[23] Spinewine A, Schmader KE, Barber N, Hughes C, Lapane KL, Swine C, Hanlon JT (2007). Appropriate prescribing in elderly people: how well can it be measured and optimised?. Lancet.

[24] Bain KT, Holmes HM, Beers MH, Maio V, Handler SM, Pauker SG (2008). Discontinuing medications: a novel approach for revising the prescribing stage of the medication-use process. J Am Geriatr Soc.

[25] Sinnott C, Hugh SM, Boyce MB, Bradley CP (2015). What to give the patient who has everything? A qualitative study of prescribing for multimorbidity in primary care. Br J Gen Pract.

[26] Anderson K, Stowasser D, Freeman C, Scott I (2014). Prescriber barriers and enablers to minimising potentially inappropriate medications in adults: a systematic review and thematic synthesis. BMJ Open.

[27] Sinnott C, McHugh S, Browne J, Bradley C (2013). GPs' perspectives on the management of patients with multimorbidity: systematic review and synthesis of qualitative research. BMJ Open.

[28] Bergert FW, Braun M, Ehrenthal K, Fessler J, Gross J, Huttner U, Kluthe B, Liesenfeld A, Seffrin J, Vetter G (2014). Recommendations for treating adult and geriatric patients on multimedication. Int J Clin Pharmacol Ther.

[29] Grimmsmann T, Schwabe U, Himmel W (2007). The influence of hospitalisation on drug prescription in primary care--a large-scale follow-up study. Eur J Clin Pharmacol.

[30] Larsen MD, Rosholm JU, Hallas J (2014). The influence of comprehensive geriatric assessment on drug therapy in elderly patients. Eur J Clin Pharmacol.

[31] Wilson A, Childs S (2002). The relationship between consultation length, process and outcomes in general practice: a systematic review. Br J Gen Pract.

[32] Muth C, Uhlmann L, Haefeli WE, Rochon J, van den AM PR, Guthlin C, Beyer M, Oswald F, Valderas JM (2018). effectiveness of a complex intervention on Prioritising multimedication in multimorbidity (PRIMUM) in primary care: results of a pragmatic cluster randomised controlled trial. BMJ Open.

[33] Muth C, Harder S, Uhlmann L, Rochon J, Fullerton B, Guthlin C, Erler A, Beyer M, van den AM PR (2016). pilot study to test the feasibility of a trial design and complex intervention on PRIoritising MUltimedication in multimorbidity in general practices (PRIMUMpilot). BMJ Open.

[34] Bosley S, Dale J (2008). Healthcare assistants in general practice: practical and conceptual issues of skill-mix change. Br J Gen Pract.

[35] Gensichen J, von Korff M, Peitz M, Muth C, Beyer M, Guthlin C, Torge M, Petersen JJ, Rosemann T, Konig J (2009). Case management for depression by health care assistants in small primary care practices: a cluster randomized trial. Ann Intern Med.

[36] Peters-Klimm F, Muller-Tasch T, Schellberg D, Gensichen J, Muth C, Herzog W, Szecsenyi J (2007). Rationale, design and conduct of a randomised controlled trial evaluating a primary care-based complex intervention to improve the quality of life of heart failure patients: HICMan (Heidelberg integrated case management). BMC Cardiovasc Disord.

[37] Rosemann T, Joos S, Laux G, Gensichen J, Szecsenyi J (2007). Case management of arthritis patients in primary care: a cluster-randomized controlled trial. Arthritis Rheum.

[38] Rosemann T, Korner T, Wensing M, Gensichen J, Muth C, Joos S, Szecsenyi J (2005). Rationale, design and conduct of a comprehensive evaluation of a primary care based intervention to improve the quality of life of osteoarthritis patients. The PraxArt-project: a cluster randomized controlled trial [ISRCTN87252339]. BMC Public Health.

[39] Gensichen J, Muth C, Butzlaff M, Rosemann T, Raspe H, de Cornejo GM, Beyer M, Harter M, Muller UA, Angermann CE (2006). the future is chronic: German primary care and the chronic care model--the comprehensive principles in the proactive treatment of the chronically ill. Z Arztl Fortbild Qualitatssich.

[40] Beyer M, Otterbach I, Erler A, Muth C, Gensichen J, Gerlach FM (2007). Multimorbidität in der Allgemeinpraxis. Teil 1: Pragmatische definition, Epidemiologie und Versorgungsprämissen. [multimorbidity in general practice. Part I: a pragmatic definition, epidemiology, prerequisites of care.]. Z Allgemeinmed.

[41] Hudon C, Fortin M, Soubhi H (2007). Abbreviated guidelines for scoring the cumulative illness rating scale (CIRS) in family practice. J Clin Epidemiol.

[42] Linn BS, Linn MW, Gurel L (1968). Cumulative illness rating scale. J Am Geriatr Soc.

[43] Fournier DA, Skaug HJ, Ancheta J, Ianelli J, Magnusson A, Maunder MN, Nielsen A, Sibert J (2012). AD model builder: using automatic differentiation for statistical inference of highly parameterized complex nonlinear models. Optim Methods Softw.

[44] Aly SS, Zhao J, Li B, Jiang J (2014). Reliability of environmental sampling culture results using the negative binomial intraclass correlation coefficient. Springerplus.

[45] Cadogan CA, Ryan C, Hughes CM (2016). Appropriate polypharmacy and medicine safety: when many is not too many. Drug Saf.

[46] Cooper JA, Cadogan CA, Patterson SM, Kerse N, Bradley MC, Ryan C, Hughes CM (2015). Interventions to improve the appropriate use of polypharmacy in older people: a Cochrane systematic review. BMJ Open.

[47] Meid AD, Haefeli WE (2016). Age-dependent impact of medication underuse and strategies for improvement. Gerontology.

[48] Rossello X, Pocock SJ, Julian DG (2015). Long-term use of cardiovascular drugs: challenges for research and for patient care. J Am Coll Cardiol.

[49] Backes JM, Ruisinger JF, Gibson CA, Moriarty PM (2017). Statin-associated muscle symptoms-managing the highly intolerant. J Clin Lipidol.

[50] Stulc T, Ceska R, Gotto AM (2015). Statin intolerance: the Clinician's perspective. Curr Atheroscler Rep.

[51] Scott IA, Gray LC, Martin JH, Pillans PI, Mitchell CA (2013). Deciding when to stop: towards evidence-based deprescribing of drugs in older populations. Evid Based Med.

[52] Furtado RH, Giugliano RP, Strunz CM, Filho CC, Ramires JA, Filho RK, Neto PA, Pereira AC, Rocha TR, Freire BT (2016). Drug interaction between Clopidogrel and ranitidine or omeprazole in stable coronary artery disease: a double-blind, double dummy**,** Randomized Study. Am J Cardiovasc Drugs.

[53] Garfinkel D, Mangin D (2010). Feasibility study of a systematic approach for discontinuation of multiple medications in older adults: addressing polypharmacy. Arch Intern Med.

[54] van der Velde N, Stricker BH, Pols HA, van der Cammen TJ (2007). Risk of falls after withdrawal of fall-risk-increasing drugs: a prospective cohort study. Br J Clin Pharmacol.

[55] Potter K, Flicker L, Page A, Etherton-Beer C (2016). Deprescribing in frail older people: a randomised controlled trial. PLoS One.

[56] Garfinkel D, Zur-Gil S, Ben-Israel J (2007). The war against polypharmacy: a new cost-effective geriatric-palliative approach for improving drug therapy in disabled elderly people. Isr Med Assoc J.

[57] Hanlon JT, Schmader KE, Samsa GP, Weinberger M, Uttech KM, Lewis IK, Cohen HJ, Feussner JR (1992). A method for assessing drug therapy appropriateness. J Clin Epidemiol.

[58] Hanlon JT, Schmader KE (2013). The medication appropriateness index at 20: where it started, where it has been, and where it may be going. Drugs Aging.

[59] Howard RL, Avery AJ, Slavenburg S, Royal S, Pipe G, Lucassen P, Pirmohamed M (2007). Which drugs cause preventable admissions to hospital? A systematic review. Br J Clin Pharmacol.

