# Supplementary figures and images for: Changes in prescribed medicines in older patients with multimorbidity and polypharmacy in general practice
Source: BMC Fam Pract. 2018 Jul 28;19:131. doi: 10.1186/s12875-018-0825-3 (PMC6064613; doi:10.1186/s12875-018-0825-3)

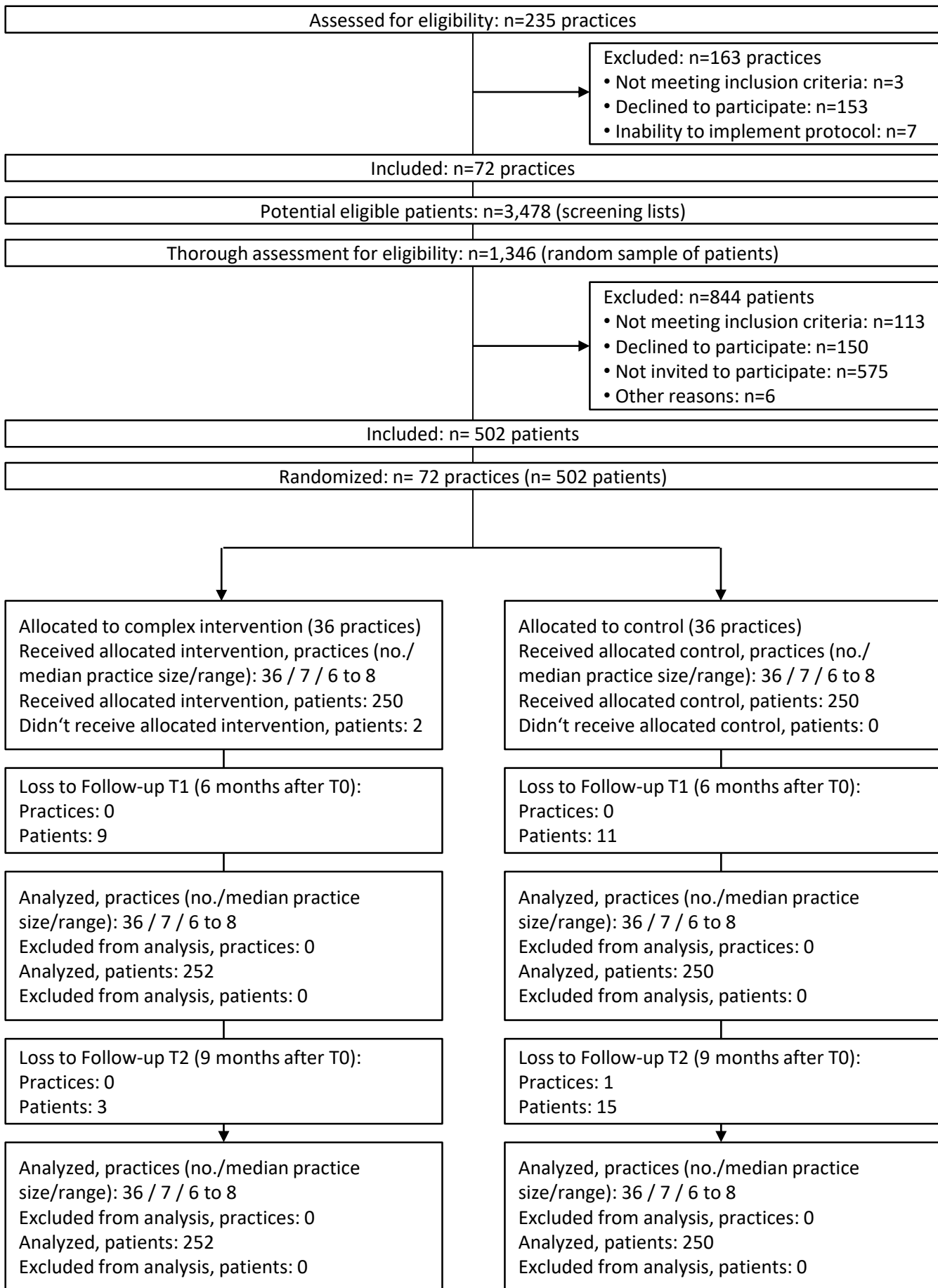

Supplement: Supplementary file 1 — Flowchart patient allocation PRIMUM Study. (PDF 340 kb) [file 12875_2018_825_MOESM1_ESM.pdf]
